# Toehold-enhanced LNA probes for selective pull down and single-molecule analysis of native chromatin

**DOI:** 10.1038/s41598-017-16864-7

**Published:** 2017-12-01

**Authors:** Nicolaas Hermans, Juriën Jori Huisman, Thomas Bauke Brouwer, Christopher Schächner, G. Paul H. van Heusden, Joachim Griesenbeck, John van Noort

**Affiliations:** 1 Leiden Institute of Physics, Huygens-Kamerlingh Onnes Laboratory, Niels Bohrweg, 2 2333 CA Leiden, The Netherlands; 2 Department of Molecular and Developmental Genetics, Institute of Biology, Leiden University, Leiden, The Netherlands; 3 Universität Regensburg, Biochemie-Zentrum Regensburg (BZR), Institut für Biochemie, Genetik und Mikrobiologie, Lehrstuhl Biochemie III, 93053 Regensburg, Germany

## Abstract

The organization of DNA into chromatin is thought to regulate gene expression in eukaryotes. To study its structure *in vitro*, there is a need for techniques that can isolate specific chromosomal loci of natively assembled chromatin. Current purification methods often involve chemical cross-linking to preserve the chromatin composition. However, such cross-linking may affect the native structure. It also impedes single molecule force spectroscopy experiments, which have been instrumental to probe chromatin folding. Here we present a method for the incorporation of affinity tags, such as biotin, into native nucleoprotein fragments based on their DNA sequence, and subsequent single molecule analysis by magnetic tweezers. DNA oligos with several Locked Nucleic Acid (LNA) nucleotides are shown to selectively bind to target DNA at room temperature, mediated by a toehold end in the target, allowing for selective purification of DNA fragments. The stability of the probe-target hybrid is sufficient to withstand over 65 pN of force. We employ these probes to obtain force-extension curves of native chromatin fragments of the 18S ribosomal DNA from the yeast *Saccharomyces cerevisiae*. These experiments yield valuable insights in the heterogeneity in structure and composition of natively assembled chromatin at the single-molecule level.

## Introduction

The activity of a gene is regulated by a plethora of proteins that contribute to structural changes in chromatin, which in turn provide access to the transcription machinery. The higher order folding of chromatin is controversial, though it is thought to play an important role in transcription regulation^1^. To elucidate chromatin-related mechanisms of transcription regulation, the structural changes and the precise stoichiometry of the proteins involved need to be resolved. This is an arduous challenge because large variations in chromatin composition have been reported within a single locus^2,3^. Moreover, temporal fluctuations further obscure compositional differences between loci^4^. A fundamental understanding of the mechanisms in transcription regulation therefore requires analysis of each genetic locus individually.

Single molecule experiments can be employed to avoid ensemble and temporal averaging of the structural properties of selected chromatin fragments. Single-molecule force spectroscopy for example has successfully resolved the composition of individual chromatin fibers, the forces that hold DNA and histones together and the interactions between nucleosomes^5–7^. Most single molecule studies on chromatin made use of synthetic DNA sequences containing regular arrays of 601 nucleosome positioning sequences, which are used to reconstitute highly homogeneous chromatin fibers *in vitro* ^8^. Such reconstituted chromatin fibers lack the variation in DNA sequence, histone composition and post-translational modifications of the histone proteins that regulate transcription. A notable exception was the pioneering study by Cui and Bustamante, who probed nucleosome interactions in natively assembled chromatin fibers from chicken erythrocytes^9^. However, these experiments were done on random fragments, impeding any insight into the local structure of a specific locus. To elucidate local chromatin structure, single molecule experiments need to be done on natively assembled chromatin on a single locus.

Extraction of a specific chromatin fragment for *in vitro* scrutiny is an enormous challenge, since a single gene represents only a very small fraction of all the chromatin present in the nucleus. Typically, an enrichment of 10^5^ or better is required for isolation of a single gene from yeast. For larger genomes, the required enrichments scale with the size of the genome. Nevertheless, several methods have been developed to meet this challenge^10^. Purification of a specific genomic region might be based on affinity purification of proteins (iChIP)^11^. A different method was developed by Griesenbeck *et al*.^12^, who cloned recognition sites for both R-recombinase and LexA binding sites into the yeast *S cerevisiae* genome next to the *PHO5* locus. Induction of recombinase and subsequent pull-down allowed for purification of a single *PHO5* promotor, which was used to reveal the nucleosome composition of the specific chromatin region. Targeting the multicopy ribosomal DNA (rDNA) locus yielded larger quantities of native chromatin, making subsequent analysis more versatile^13^.

Alternatively, modified nucleic acids like Peptide Nucleic Acids (PNA) or Locked Nucleic Acids (LNA), in combination with affinity tags, have been used to sequence-specifically pull down DNA^14,15^. In LNA nucleotides, the methylene bridge between the 2′ oxygen and the 4′ carbon forces the sugar into a locked C3′ endo configuration, which is favorable for base stacking^16^, increasing the melting temperature by up to 5 °C per substituted nucleotide^17^. Applications of LNAs include single-molecule manipulation experiments^18^, and enhancing the specificity of RNA/DNA aptamers^19^. LNA oligos have for example been employed to target telomeric chromatin in nuclear extracts^15^, and later to target ribosomal DNA^20^. In these studies, LNA invaded the target DNA duplex, which required high temperatures (80 °C) for effective hybridization. To maintain the compositional integrity of chromatin fragments during this pull-down, chemical crosslinking of the chromatin was necessary. Especially for the analysis of higher order structures of chromatin it would be preferable to refrain from such fixation, because it has been implied to induce structural changes^21^.

As a generic method to obtain specific native chromatin fragments, without the need for genetic modifications, we designed biotinylated LNA probes that can invade and hybridize double stranded (ds) DNA at temperatures below 37 °C. A biotinylated nucleic acid hairpin, including an 18-base single-strand overhang containing 6 LNA bases, is shown to capture DNA fragments in a sequence specific manner. Rather than elevated temperatures and crosslinking, we use restriction enzymes to create a 4-base toehold on the target DNA which enhances both hybridization efficiency and specificity. We used these LNA probes to insert a digoxigenin (Dig) tag on the 18S fragment of yeast rDNA, and subsequently to tether the molecules between a paramagnetic bead and an antibody coated glass coverslip.

For the single molecule analysis of these chromatin fragments, magnetic tweezers are particularly convenient since the magnetic beads can be used both for a selective pull down and for force spectroscopy. Moreover, magnetic tweezers have a relatively high throughput and can be multiplexed^22^, which makes it feasible to measure hundreds of beads in parallel. Here we demonstrate the use of single molecule force spectroscopy to probe the mechanical properties of single chromatin fibers from the 18S rDNA locus. This is an important step forward for single molecule force spectroscopy methods, as it shows for the first time the structure, dynamics and compositional variations of a specific, natively assembled chromatin fragment, paving the path for a detailed structural analysis of real chromatin including its natural DNA sequence, histone composition and patterns of histone modifications.

## Results

### Probe design

**Figure 1:**
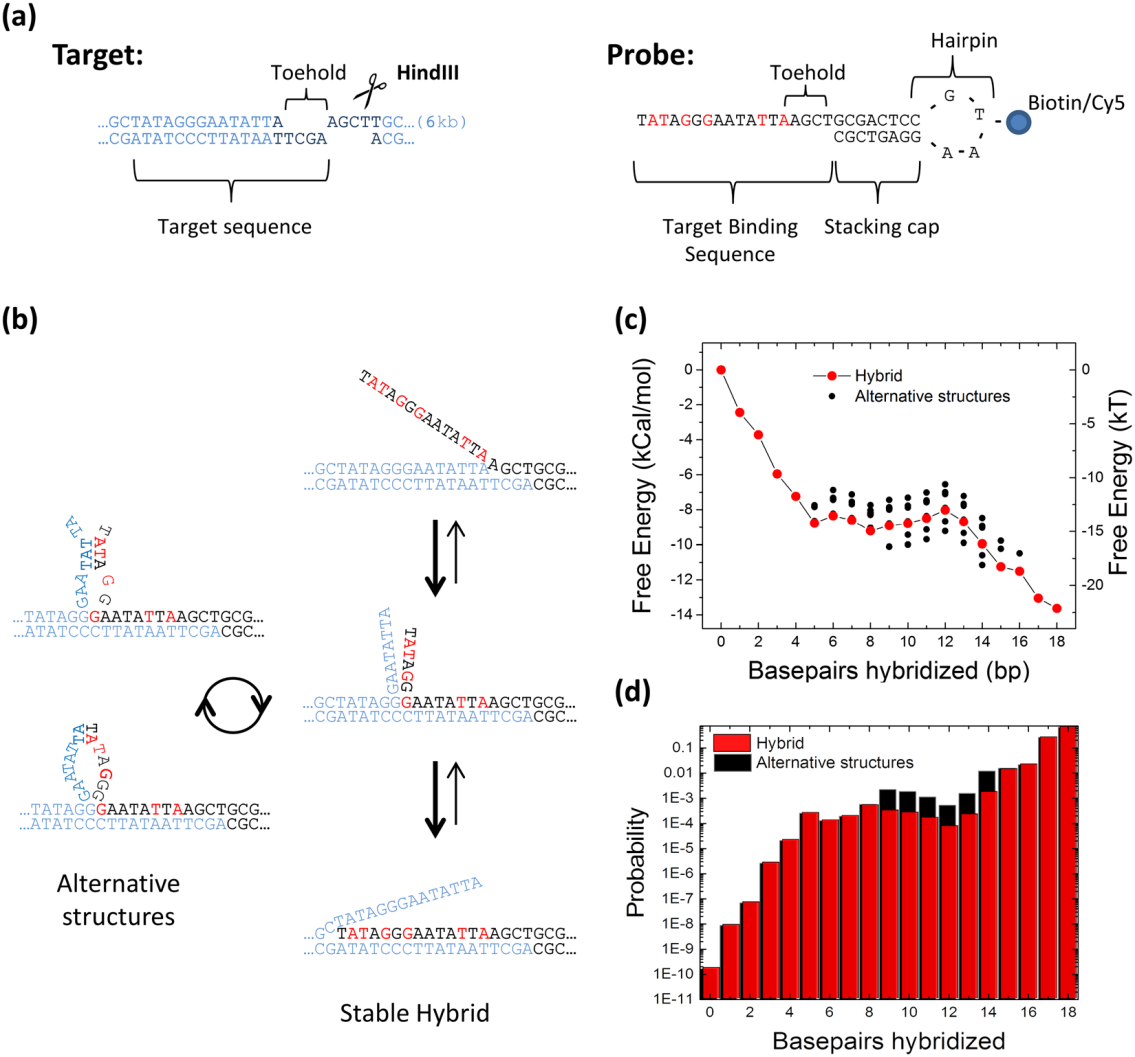
Rational probe design results and high predicted affinity. (**a**) A target sequence, with *Hind*III restriction site (bold) resulting in a toehold, and the complementary LNA-probe. LNA nucleotides are red, DNA nucleotides black. (**b**) Schematic representation of the strand invasion. First, the probe binds the toehold on the target. Fraying of the DNA ends allows for strand invasion, and finally a stable hybrid is formed. In addition, secondary structures can form between the displaced target strand, and un-incorporated nucleotides from the probe, resulting in alternative structures. Two alternative structures with 12 bases incorporated are shown on the left. (**c**) Calculations of the free energy change of hybridization, as the number of hybridized nucleotides of the probe increases. The red line depicts the hybrid, in which the target strand hybridizes linearly with the probe. Black dots indicate alternative LNA-DNA hybrids between the displaced strand and the probe. (**d**) Alternative structures can stabilize intermediate structures. In equilibrium the full hybrid accounts for 87% of the hybridization products, calculated by the Boltzmann probability distribution.

Efficient pull-down of natively folded chromatin requires a probe that has i) a high affinity for the target, ii) a sufficiently high hybridization rate at temperatures that maintain proteins in their folded, DNA bound state and iii) a high specificity, e.g. have a much lower affinity for non-complementary sequences. In order to satisfy these criteria, we designed an affinity tagged nucleic acid hairpin containing an 18-base overhang at the 5′ end that is complementary to the T7 promotor sequence in the pYES2 plasmid (Fig. 1a). To maximize the binding affinity, we included several LNA nucleotides in the DNA oligo, referred to as the LNA probe. Previously, it was shown that LNA probes have a higher efficiency when invading DNA ends^20^. By cleaving pYES2 with the restriction enzyme *Hind*III, a 4 base overhang directly adjacent to the target sequence was created. The 5′ overhang in the target forms a toehold that facilitates the initial binding of the LNA probe (Fig. 1b). When the probe-toehold hybrid is formed, the free 5′ end of the target can stack onto the 3′ bases in the hairpin, which further stabilizes the initial complex. The spontaneous fraying of the ends of the DNA target duplex at room temperature is expected to facilitate the invasion of the dsDNA target by the remaining part of the LNA probe. The binding affinity of the single stranded LNA probe for the target exceeds the affinity of the anti-sense strand of the target itself, forming a stable LNA-DNA hybrid.

To get more insight in the design strategy for optimal LNA probes, we used a nearest-neighbor model for DNA and LNA nucleotides to calculate the energy difference between the probe and the target-probe hybrid^17,23^. From this model, the Gibbs free energy change for the hybridization reaction is plotted per hybridized base in Fig. 1c. The melting of the target duplex over the required length (14 bp) involves a net free energy increase of 12.2 kCal mol^−1^ (Fig. 1c). Subsequent hybridization of the probe with the target strand decreases the free energy by 23.4 kCal mol^−1^, which involves 5.9 kCal mol^−1^ for hybridization of the 4-nucleotide toehold and 17.5 kCal mol^−1^ for hybridization with the displaced strand. Basepair stacking of the free 5′ end of the target to the 3′ end of the probe further decreases the free energy with 2.4 kCal mol^−1^. This results in a net free energy change of −13.6 kCal mol^−1^ (Fig. 1c), corresponding to an affinity for the probe-target hybrid of K_d_ = 0.2 nM. As shown in Fig. 1b, a plethora of reaction intermediates and alternative hybridization products can also be envisioned. When intermediates featuring base-stacking of the partially displaced strand with the partially unbound probe are included, statistical physics suggests that the structure in which the full length of the probe is hybridized accounts for 87% of all products (Fig. 1d).

### Probe specificity

**Figure 2:**
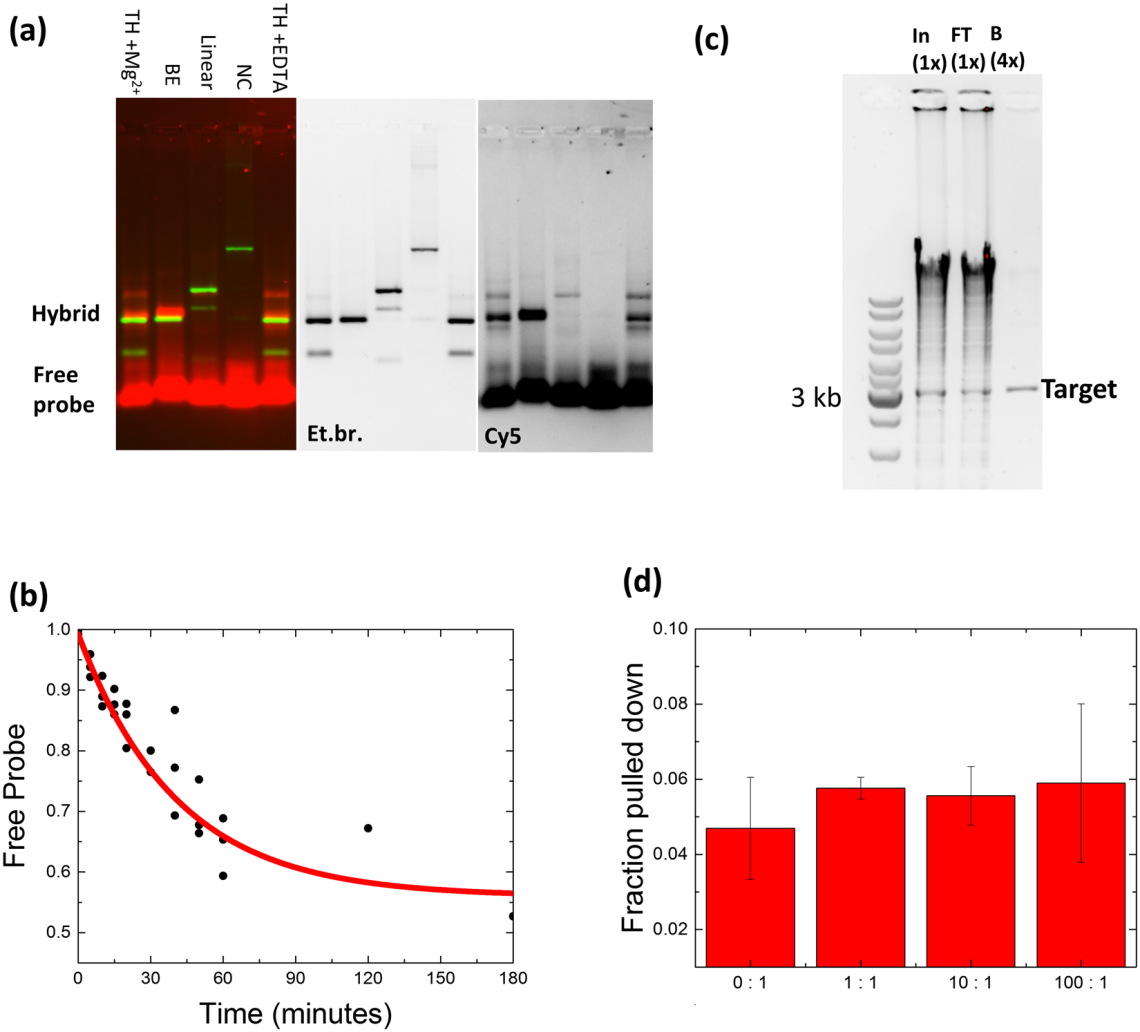
Sequence specific hybridization of an LNA probe with the target sequence. (**a**) The LNA probe has increased binding for a target with a compatible toehold. The Cy5 labeled pLNA-1 was hybridized to different targets, TH: target with toehold, in the presence of either 10 mM Mg^2+^ or 10 mM EDTA, BE: blunt end target including toehold sequence, Linear: DNA fragment with the target sequence at 116 bp from the end; NC: DNA fragment without the target sequence (Sequences in Supplementary Table S1). After hybridization the DNA was separated in a 2% agarose gel and visualized with Ethidium bromide (EtBr, green) and the Cy5 label (red) (left panel, composite). The separate Ethidium bromide and Cy5 channels are shown on the right. (**b**) Hybridization of the LNA probe (5.6 nM) and target (56 nM) over time, measured by gel electrophoresis (see Figure S1). Data was fitted with a single exponential decay. The offset corresponds to K_*d*_ of 39 nM, yielding a half-life for the unbound probe of 60 minutes. (**c**) Genomic DNA from *E.coli* digested with *Hind*III was mixed with target DNA in a 100:1 ratio, and subjected to purification using an LNA toehold as described in Methods. DNA contained in samples from input (In), flow through (FT), and recovered from beads (B) was separated in an agarose gel and visualized with EtBr. The relative fraction of each sample loaded is indicated on top. Positions of selected marker bands in the first lane and the target are shown on the left and right, respectively. (**d**) Quantification of the recovery of the DNA target from different mixtures with varying competitor (genomic DNA from *E. coli*) to target ratios.

We tested the ability of a Cy5 labeled fluorescent LNA-probe to bind to four different DNA targets at 37 °C within 1 hour (Fig. 2a, Sequences in supplemental material Table S1). The optimal substrate contains both the target sequence and the toehold. There are some additional bands, tentatively attributed to alternative secondary structures of target-probe hybrids (Figure S1). Overall, Fig. 2a shows the high sequence specificity and the importance of the toehold to facilitate target strand invasion by the LNA probe.

To investigate the kinetics of target invasion by the probe, we measured a time series at 37 °C (example agarose gel shown in Supplementary Figure S1). Figure 2b shows the decay of the concentration of free probe, compared to the amount of hybridized product as determined from the fluorescence intensity of the corresponding bands in the gel. An exponential fit yields a decay time of 60 ± 10 min (mean ± S.E.) and an equilibrium constant, K_*d*_ = 39 ± 9 nM (mean ± S.E.). Though this affinity is lower than the calculated affinity, it is sufficiently strong for pull-down of the target (see below). The presence of alternative structures, as shown in Figure S1, results in more complex thermodynamic behavior that cannot be captured in a single affinity constant, which may explain this discrepancy. Nevertheless, a decay time of the free probe of about an hour is reasonable for practical experiments, in which chromatin integrity needs to be maintained during sample preparation.

We tested the ability and efficiency of the LNA probe to selectively pull down a specific DNA fragment from a mixture containing an excess of competitor DNA consisting of genomic DNA from *E.coli* K12, digested with *Hind*III. The *E.coli* genome contains 556 *Hind*III restriction sites, yielding 1112 toeholds that are compatible with our probe. However, none of these fragments contain a perfect match for the LNA overhang; sequences adjacent to the *Hind*III site have an overlap of up to 9 bases (Supplementary Figure S2). From a mixture of 100 ng of *Hind*III digested pUC18 containing the target sequence and up to 10 μg genomic DNA, about 5% of the target can be recovered by magnetic beads based pull-down, after a hybridization of 1 hour (Fig. 2c,d). Most of the lost target DNA, about 90%, is found in the flow through. Compared to hybridization using fluorescent probes, we observed a lower yield in the pull-down. Steric hindrance of the paramagnetic bead may decrease the probe-bead hybridization efficiency. Consistent with this, the ability to pull down DNA is independent of competitor DNA concentration (Fig. 2d) or the sequence of the toehold in the competitor DNA (Supplementary Figure S3). Importantly, we observe almost no competitor DNA after two wash steps, showing the highly specific nature of the LNA probes.

### LNA probes for Single-Molecule Force Spectroscopy

**Figure 3:**
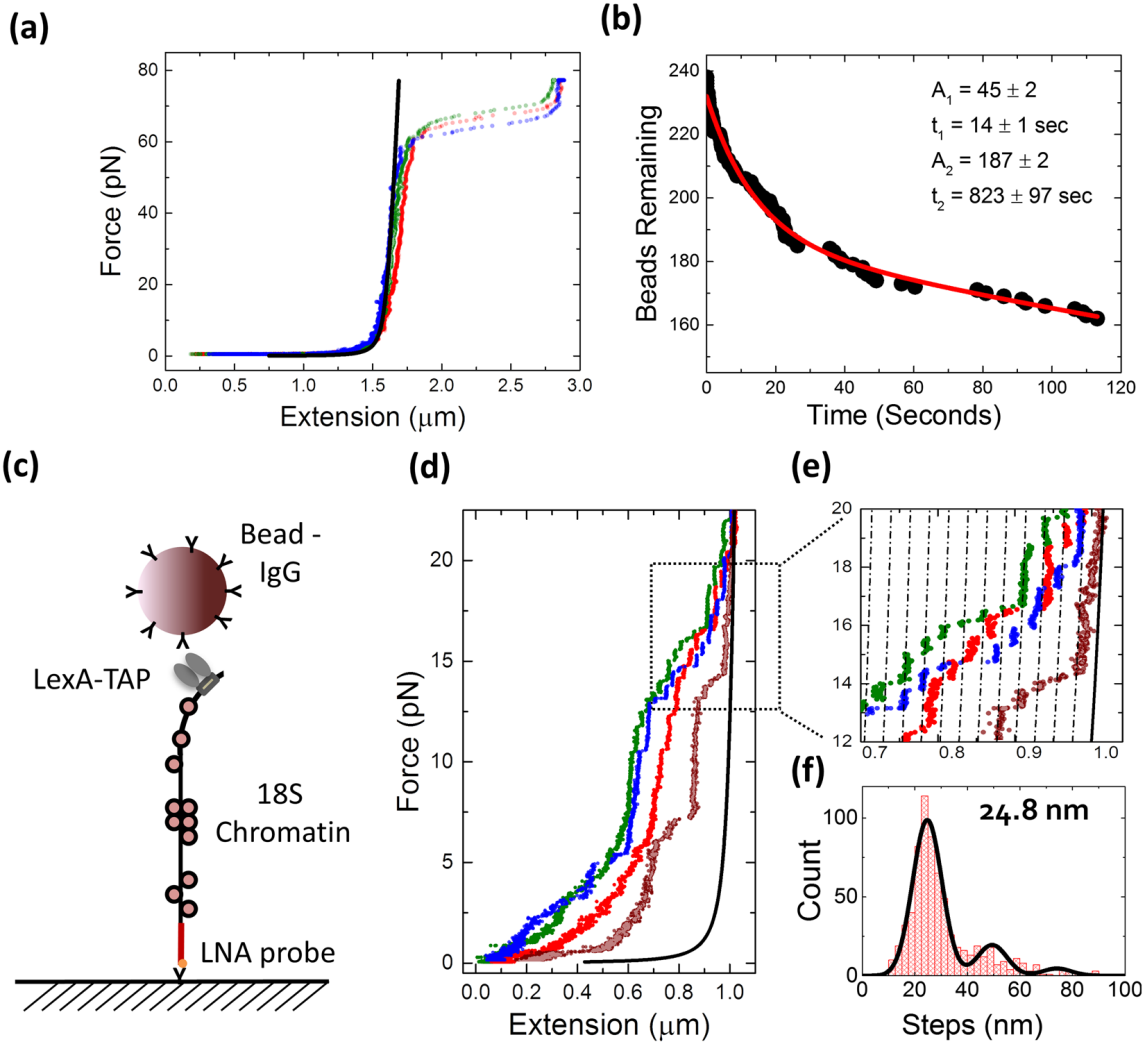
The use of LNA toehold probes in magnetic tweezer experiments. (**a**) Force-extension curves of 4.8 kb DNA tethered with an LNA toehold to the glass surface, showing the overstretching plateau at around 65 pN. Of these beads 44% ruptured before overstretching, 22% during the 4 seconds of overstretching, and 34% of the tethers stayed intact after overstretching (*N* = 176). The black line represents a worm-like chain (WLC) model for DNA of 4.8 kb, and 3 individual molecules are shown in color. (**b**) Rupture of beads at 35 pN was fit with a double exponential decay. A total of 238 beads were monitored, and 76 ruptures were detected within 2 minutes. (**c**) Schematic representation of an 18S chromatin molecule immobilized on the glass surface by the LNA toehold probe and 3 LexA-TAP proteins to an IgG coated bead. (**d**) Force extension curves of native chromatin. Each curve represents a single fragment isolated from the yeast extract. (**e**) Discrete steps in extension of 25 nm (dotted lines), characteristic for the unwrapping the DNA of the inner turn from a single nucleosome. (**f**) Histogram of the steps of the inner turn unwrapping and fit, yielding a step size of 24.8 ± 0.1 nm (mean ± S.E.M.).

To assess the use of LNA-toehold probes for single-molecule force spectroscopy, we coupled DNA fragments with a biotinylated LNA probe to streptavidin coated paramagnetic beads. Using standard magnetic tweezers protocols, the other end of the DNA was ligated to a PCR product containing multiple Digoxigenins that bind to the anti-Digoxigenin coated glass surface of a flow cell. We subsequently obtained force-extension curves of the tethered DNA molecules (Fig. 3a). As expected, the force-extension behavior of the DNA closely follows a worm like chain (WLC) model at forces below 65 pN. Importantly, the LNA-toehold can endure forces exceeding 65 pN, shown by the characteristic overstretching plateau, where the two strands melt^24,25^. Out of 176 tethers, 56% remain intact beyond the overstretching plateau at 65 pN. About half of these tethers (22% of the total) ruptured within 2 seconds after the overstretching transition occurred. For comparison, DNA hybrids of similar length (<20 basepairs) show rupture forces ranging from 20 to 50 pN, at a pulling rate of 500 pN s^−1^ ^26,27^. Our pulling rate was significantly smaller (~5 pN s^−1^), which underscores the superior stability of LNA-DNA hybrids over dsDNA.

To better assess the force limitations of the LNA probe-target hybrid, we pulled at a constant force of 35 pN for 2 minutes, recording each rupture event (Fig. 3b). Out of 240 beads, 167 beads remain tethered after 2 minutes. The rupture events show a bi-exponential behavior, where 81% features a decay time of 823 ± 97 seconds, while 19% of the tethers rupture with a half-life of 14 ± 2 seconds. If we assume all these rupture events are caused by disruption of the DNA-LNA hybrid, this bi-exponential behavior suggests that the LNA probes hybridize in at least 2 conformations, which results in different stabilities. Nevertheless, the majority of the tethers can withstand forces of several tens of pN for minutes, making the LNA-toehold probes suitable for most force spectroscopy applications.

### Force spectroscopy on native chromatin fibers

We tested whether LNA-toehold probes could be used to introduce affinity tags into natively folded chromatin from a specific locus. Chromosomal fragments from the 18S rDNA locus from the yeast *S. cerevisiae* were tethered to magnetic beads using a LexA based fusion protein with a tandem affinity tag as described earlier^28^. Rather than using the calmodulin purification as second step to further purify the substrate, we cut the chromatin fragments with the restriction enzyme *Ava*I and hybridized a LNA-toehold probe containing a single Dig, which is complementary to the sequence adjacent to the *Ava*I site (Fig. 3c). These chromatin fragments were subsequently tethered on the anti-Dig coated surface of a flow cell. The LNA probe serves as second affinity tag for purification as well as immobilization anchor for the force spectroscopy. Multiple force-extension curves of 18S rDNA chromatin fibers are shown in Fig. 3d. We aligned the maximal extension of the 18S rDNA chromatin with a WLC model of 3041 bp DNA, corresponding to the force-extension curve of the bare 18S fragment. At 20 pN we expect all the nucleosomes to be fully unwrapped. Indeed, the extension of the chromatin at forces exceeding 20 pN overlaps with the corresponding WLC, confirming that tethers are 18S rDNA fragments.

One of the characteristic force-induced transitions in chromatin is the unwrapping of the inner turn of DNA from the histone core, showing steps of approximately 25 nm^29,30^. Indeed, such steps in extension are also clearly resolved in the force-extension curves of the 18S rDNA fragment (Fig. 3e). The average step size for the steps above 5 pN is 24.8 ± 0.1 nm, for 312 steps obtained from 29 chromatin fibers (Fig. 3f). Given the characteristic step size and rupture force, we can attribute each step to a single nucleosome. For the 4 molecules shown in Fig. 3d, the number of nucleosomes varies between 9 and 14. Since some of the 18S rDNA regions may be actively transcribed, while others are transcriptional inactive^31^, and transcription has been implicated to induce partial depletion of nucleosomes, a large variation in the number of nucleosomes on these molecules is to be expected. By counting the number of steps, we can thus determine the number of nucleosomes on individual chromatin molecules, exploiting one of the advantages of single-molecule analysis, i.e. the ability to resolve heterogeneities within a population.

Finally, we tethered the 18s rDNA chromatin fiber by targeting LNA-toehold probes to both ends of the 18S fragment, where one probe contained a single biotin and the other a Dig. The genetically encoded LexA sites were only used for purification and not for immobilization (Supplementary Figure S4). Chromatin tethered with two LNA probes resisted higher forces than LexA tethered chromatin fibers; only 12% of double LNA tethers ruptured at forces below 25 pN (*N* = 188), while 52% of the tethers immobilized with LexA and LNA ruptured below 25 pN (*N* = 130). Using dual LNA probes thus increased stability of the tether, while also presenting a more generic, flexible method for chromatin tethering.

## Discussion

In this work, we show that LNA-toehold probes can be used to introduce affinity tags into DNA fragments in a sequence specific manner, without the need for temperature-induced melting of the DNA. The LNA nucleotides in the probe are shown to increase the binding affinity enough to be able to outcompete the complementary strand in the target DNA. In a similar fashion, Smolina *et al*. showed the inability of PNA oligonucleotides to invade internal sites of a double helix, whereas end-duplexes were readily formed^14^. LNA probes were also shown before to invade DNA ends more readily^20^, but the addition of a small toehold as we show here, speeds up the reaction significantly and allows for hybridization at lower temperatures. A fast on-rate is important for applications with delicate biological samples, which deteriorate over time at ambient temperatures because of protein denaturation and degradation of proteins and DNA. This makes the approach described here suitable for non-cross-linked samples, in contrast to previous reports on specific pull-down of nucleoprotein complexes^11,15,20^.

Due to the high affinity of the LNA-toehold probe for the target sequence, the LNA-DNA hybrid can sustain forces over 65 pN for several seconds. At a constant force of 35 pN, we observed stable tethering for 81% of the molecules for 2 minutes. This is substantially higher than the rupture forces of DNA hybrids that were reported before, such as the 17–40 pN needed to rupture a 12 base DNA oligo^27^, and 10–15 pN for the 8 nucleotides in Lambda DNA^32^. LNA-DNA triplet structures of 15 nucleotides were shown to withstand shear forces of 12 pN^18^. The higher rupture force of our LNA-DNA hybrids could be attributed to the higher affinity of the LNA nucleotides to their DNA counterparts, and the longer 18 bp hybridization length. A bi-exponential decay of the survival probability of the tethers suggests at least 2 populations of hybrids. Short-lived ruptures could be caused by alternative structures, when the probe is not fully hybridized to the target. According to a nearest-neighbor model calculation, the fully displaced hybrid should account for 87% of the hybrids, which corresponds reasonably well with the 81% of high-force resistant tethers. Taken together, the affinity and force resistance make LNA-toehold probes particularly useful for single molecule force spectroscopy on protein-DNA complexes.

We show here that LNA-toehold probes can be used directly to immobilize partially purified native chromatin fragments from cell extracts for single molecule manipulation experiments. The native chromatin force-extension curves give insight in the compositional heterogeneity that occurs at a single chromatin locus. A large heterogeneity is to be expected, since the state of compaction can be different at each of the 150 rDNA loci within a single cell^33^. Many of these loci are actively transcribed. Mass spectrometry on the full 35S fragments shows that some of the RNA polymerases are still attached to the chromatin fragments^28^. Here, we did not observe any signatures of RNA polymerase or any other proteins, except for histones. Refraining from any fixation of the chromatin, and diluting the chromatin fragments to low concentrations may lead to dissociation of many of the proteins that were initially bound to the DNA. However, it is also possible that these proteins do not show a characteristic feature in the force-extension curve, or that such features are hidden in the low-force part of the curve. Further analysis of the non-cross-linked chromatin fragments, for example by mass spectrometry or by microscopy using fluorescent anti-bodies, may shed more light on this.

Nevertheless, characteristic features of chromatin can be observed. The 25 nm steps in extension at higher forces correspond exactly to the unwrapping of the inner turn DNA in the nucleosome, which was shown previously for fibers reconstituted with purified histones^29^. The forces at which these steps occur are also in the same range as measured on reconstituted fibers under the same buffer conditions. For reconstituted chromatin, the number of these 25 nm steps corresponds to the number of nucleosomes on the DNA^29,34^. For native chromatin, it is unlikely that other structures are responsible for releasing about 75 bp of DNA, facilitating the interpretation of the curves.

The higher order structure of chromatin fibers is a highly-debated topic to which force spectroscopy can contribute^34^. Deeper insight in the folding of native chromatin fibers requires a more intricate analysis that goes beyond the scope of this paper. Whereas the second turn unwrapping of a nucleosome will proceed independently of others, breaking interactions between nucleosomes obviously depends on the presence and location of multiple nucleosomes. As opposed to the regularly spaced nucleosomes on 601 arrays^34^, natively assembled nucleosomes are expected to be irregularly spaced^35^, which will lead to a complex unfolding profile at lower forces. In addition, the H2A-H2B dimers that are indispensable for nucleosome-nucleosome interactions are more fragile than the H3-H4 tetramers that constrain the inner wrap of DNA around the nucleosome core, and some of the dimers may dissociate during purification. Nevertheless, we observed a significant level of DNA condensation at forces below 5 pN, showing that at least a part of the higher order structure survives the pull-down protocol without crosslinking. Further analysis of the single molecule experiments on chromosomal fragments in their native state is expected to provide more insights in the composition and higher order structure of native chromatin.

Using our method, it is possible to maintain the diverse composition of chromatin fragments from the nucleus of the cell, which allows capitalizing one of distinguishing features of single-molecule analysis: the power to resolve variations within a population. LNA-toehold probes make it practically feasible to extract and manipulate specific chromatin fragments directly from cell extracts, which is a major step forward in determining how the compositional and structural heterogeneity in chromatin can drive transcriptional regulation.

## Methods

### LNA probes and constructs

LNA probes pLNA1 (5′-tATaGgGaataTtAagctgcgactccg-t*-aaggagtcgc-3′, where LNA nucleotides are capitalized, and t* is a either a biotin-dT or Cy5-dT), pLNA2 (5′-cAgTgaTaTGatcTcggcggatccgctt-t*-ttgcggatccg-3′, t* is a Dig-dT) and pLNA3 (5′-cTaGAgtcgacCcGacctgcaggctt-t*ttgcctgcagg-3′, t* is a biotin-dT) were purchased from Eurogentec. The number of LNA nucleotides that can be incorporated is limited by the sequence of the probe, as oligos with a high LNA content are prone to self-hybridization^36^. Self-hybridization was checked with the LNA oligo design tool available on the Exiqon website (https://www.exiqon.com/ls/Pages/ExiqonOligoOptimizerTool.aspx). The location of the LNA nucleotides was selected such that self-hybrids had a melting temperature below 37 °C. To test hybridization efficiencies, the target sequences were cloned into the pBluescript SK(+) multiple cloning site by a restriction digestion with *Xba*I and *Hind*III and ligating in the following adapter: 5′-tcgactatagggaatattaagcttctagagtcgacccgagatcatatcactgg-3, 5′-ccggccagtgatatgatctcgggtcgactctagttgc-ttaatattccctatag-3′.

### K_d_ and hybridization rates

The T7 promoter region from pYES2 was amplified by PCR and cut with *Hind*III to generate a compatible toehold for pLNA1 (sequence of the resulting target in supplementary material Table S1). A total of 5.6 pmol of target and 0.56 pmol of pLNA1-Cy5 were mixed and incubated at 37 °C (final volume of 100 µL) in NEBuffer 2 (New England Biolabs). Samples were taken at the indicated time intervals and separated on a 3% agarose gel with 0.2 mg/ml ethidium bromide in 0.5x TBE, supplemented with 1 mM MgCl_2_. All gels were quantified using ImageJ software. For each lane, the signal corresponding to the free probe and hybrid was corrected for the local background and normalized to the cumulative signal of both the probe and hybrid within each lane (Figure S1). The *K*_*d*_ was determined from the average of single-exponential decay fits of 3 independent experiments. For the hybridization in Fig. 2a, all samples were hybridized for 60 minutes at 37 °C in a buffer containing 100 mM NaCl, 10 mM Tris pH 7.5 and 10 mM MgCl, except for the last lane where the MgCl was omitted and 10 mM EDTA was added instead.

### DNA Pull-Down

*E. coli* chromosomal DNA (XL-1 Blue) was obtained by phenol extraction following ethanol precipitation of an overnight culture grown in 250 ml of LB medium. DNA was digested with either *Hind*III or *Ava*I (New England Biolabs) to create appropriate toeholds. For the pull-down, 100 ng target DNA was mixed with 10 µg genomic DNA in a total volume of 50 µL in NEB buffer 2 (New England Biolabs). LNA probe was added to a final concentration of 200 nM, and incubated at 37 °C for 1.5 hours. Subsequently 10 µg of streptavidin coated M280-dynabeads (ThermoFisher Scientific) was added and incubated for 5 minutes on ice. Beads were washed with NEB buffer 2.1 up to 5 times. When required, DNA was released from the beads by adding 2 times excess of an oligo complementary to the overhang of the LNA probe (5′-agcttaatattccctata-3′ for pLNA1 and 5′-ggtcgggtcgactctag-3′ for pLNA3) and heating at 70 °C for 20 minutes, followed by a slow cool down to room temperature. Samples were loaded on a 1% agarose gel in 0.5x TBE buffer, supplemented with 0.2 mg/ml ethidium bromide.

### Force spectroscopy

To test the maximal force that the LNA toehold can withstand, 1 µg pYES2 plasmid was digested with *Hind*III and *Bsp*HI (New England Biolabs). A 1200 bp PCR product with 5% ddATP-DIG was made as described previously^37^, and also digested with *Bsp*HI to create two fragments of ~600 bp. After heat-inactivating the enzymes for 20 minutes, the pYES2 target DNA and the PCR product were ligated with T4 ligase for 1 hour at room temperature. T4 ligase was heat inactivated at 70 °C for 20 minutes. LNA probe (pLNA2dig) was added to a final concentration of 400 nM, and hybridized over 1 hour at 37 °C. Subsequently, 10 ng of DNA and 10 µg of streptavidin coated paramagnetic beads (M270 Dynabeads, ThermoFisher Scientific) were added to 500 µl of measurement buffer (MB: 10 mM Hepes pH 7.5, 150 mM KCl, 0.2% BSA, 10 mM NaN3, 0.2% Tween20). Magnetic tweezer experiments were done as described previously^37^. A maximal force of 80 pN was applied in the DNA overstretching measurements. A constant force of 35 pN was applied for 2 minutes for the rupturing experiments. The distribution of rupture times was fitted with a double exponential decay:

1 $$y(t)={{A}_{1}}^{-\frac{t}{t1}}+{{A}_{2}}^{-\frac{t}{t2}},$$

where *y* is the total number of tethered beads, *t* is the time in seconds, *A*_1_ and *A*_2_ are fraction sizes of the two observed populations, and *t*1 and *t*2 are the rupture times for each fraction. At a constant force of 35 pN, fitting yielded *A*_1_ = 45 ± 2, *t*1 = 14 ± 1 s, *A*_2_ = 187 ± 2, *t*2 = 823 ± 97 s.

### Purification of native Chromatin

Yeast cells (strain Y3280) were grown and induced as described previously^13^. Cells were flash frozen in liquid nitrogen and ground with dry ice in a coffee grinder. The resulting powder, containing both ground yeast cells and dry ice, was stored in a 50 ml tube at −80 °C until further use. IgG was coupled to epoxy coated paramagnetic beads (M270 Dynabeads, ThermoFischer Scientific) following manufacturer’s instructions. Before an experiment, 50–200 µg of ground yeast was slowly thawed on ice with 200 µl of buffer W (20 mM Tris pH 8.0, 200 mM KCl, 5 mM MgCl, 0.5% TritonX-100, 0.1% Tween20, 0.2 mg/ml Benzanidine, 1 mM PMSF, 10 mM DTT). After centrifugation at 14.000 g for 30 minutes at 4 °C, 50–200 µg of IgG coupled beads were added to the cleared supernatant. After 5 minutes of incubation on ice, beads were washed 5 times in buffer W. The buffer was exchanged to 1x NEB cutsmart buffer (New England Biolabs) after the final wash step, supplemented with 40 units of *Ava*I and 400 nM pLNA2-bio probe (final volume of 50 µl). After 2 hours at room temperature or ~48 hours at 4 °C, 2–10 µL sample was added to 500 µL buffer MB complemented with 2 mM MgCl_2_ (final concentration), and flushed into the flow cell.

For the double LNA experiments, chromatin fragments were purified using only the calmodulin resin as described earlier^28^. The equivalent of 0.3 pmol of sample was cut with *Ava*I in a volume of 50 µL, and both pLNA2-Dig and pLNA3-Bio were added to 400 nM final concentration. Hybridization was carried out 4 °C for 48 hours, and 5–10 µL sample was mixed with 500 µL MB and 5 µg streptavidin coated paramagnetic beads with a diameter of 1.4 µm (Microparticles) or 2.8 µm (M270 Dynabeads, ThermoFischer Scientific).

Force-extension curves were analyzed using a step finder function as described in Meng *et al*.^34^, using a windowed median filter instead of a mean filter. The time window was varied between 5 and 10 points to optimize performance for individual curves. The resulting distribution is plotted in Fig. 3f. The cumulative distribution was fit with the CDF of three normal distributions;

2 $$y={A}_{1}\{1-erf(\frac{x+step}{\sigma \sqrt{2}})\}+{A}_{2}\{1-erf(\frac{x+2\,\ast \,step}{\sigma \sqrt{2}})\}+{A}_{3}\{1-erf(\frac{x+3\,\ast \,step}{\sigma \sqrt{2}})\},$$

where *step* is the step size, $\sigma$ is the standard deviation and *A*_1_, *A*_2_ and *A*_3_ are the amplitudes of the normal distributions, corresponding to the simultaneous unwrapping of 1, 2 or 3 nucleosomes. An average step size of 24.8 ± 0.1 nm was obtained, with a standard deviation of 5.5 nm. Corresponding normal distributions were overlaid to the histogram in Fig. 3f.

For the rupturing analysis the fraction of tethers ruptured at 25 pN was recorded. To discard non-specifically tethered beads, we selected tethers that had an extension in the range of 900–1200 nm between 10–20 pN, corresponding to the contour length the 18S rDNA fragment. For 130 chromatin fibers tethered with LNA and LexA, 67 ruptured below 25 pN (52%). Out of 188 tethers immobilized with LNA on both sides, 23 ruptured below 25 pN (12%), demonstrating enhanced stability of LNA probes over LexA immobilization.

### Data availability

Data corresponding to the main figures is made available in the supplementary material.

## Electronic supplementary material

Supplementary Information
