# Supplementary material for: Toehold-enhanced LNA probes for selective pull down and single-molecule analysis of native chromatin
Source: Sci Rep. 2017 Dec 1;7:16721. doi: 10.1038/s41598-017-16864-7 (PMC5711847; doi:10.1038/s41598-017-16864-7)
Supplement: Supplementary file 1 — Supplementary Information [file 41598_2017_16864_MOESM1_ESM.pdf]

Toehold-enhanced LNA probes for selective pull down and single-molecule analysis of native chromatin

Nicolaas Hermans, Juriën Jori Huisman, Thomas Bauke Brouwer, Christopher Schächner, G. Paul H. van Heusden, Joachim Griesenbeck and John van Noort

Supplementary Information

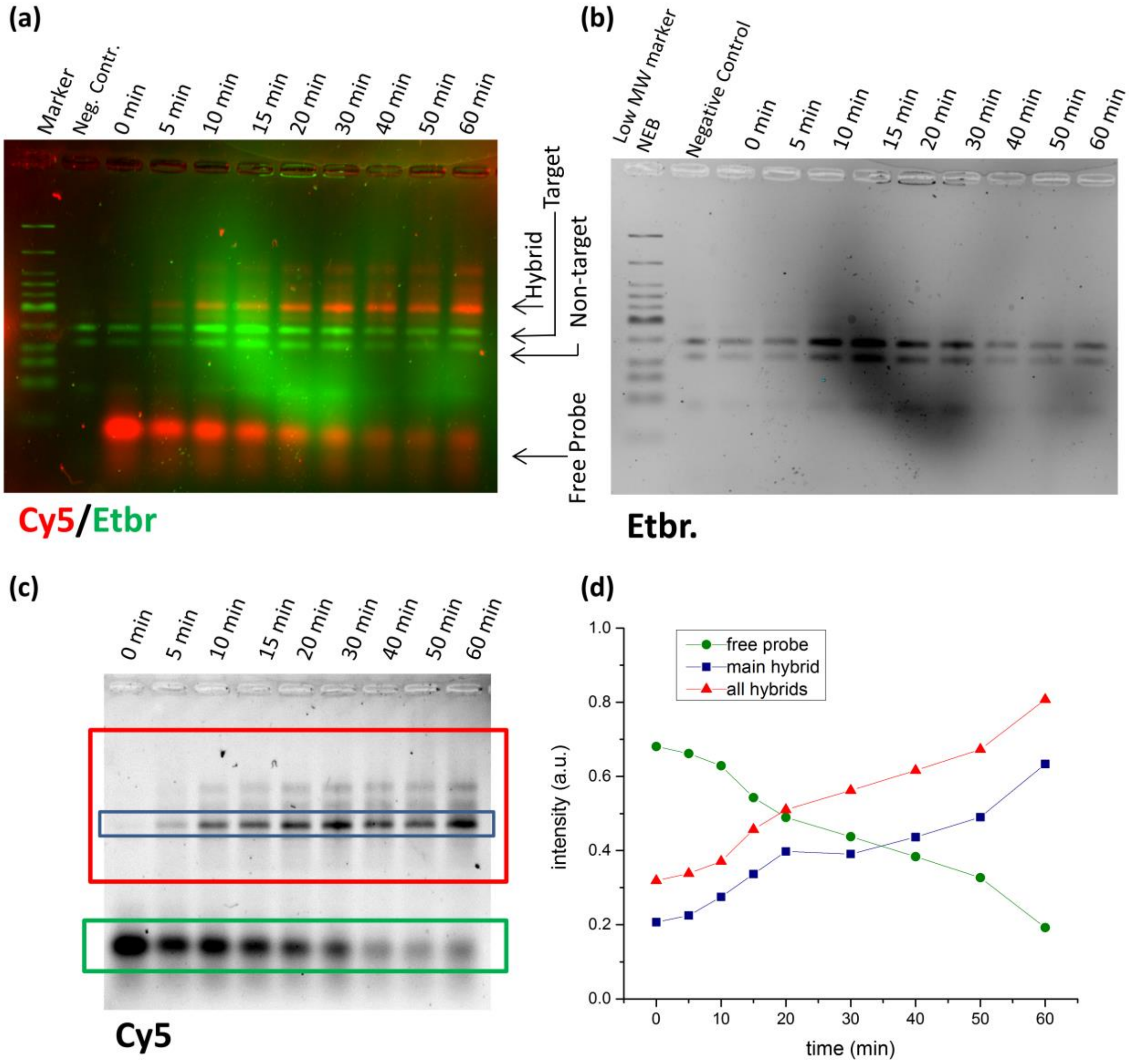

Figure S1: Hybridization of the LNA probe into the target over time at 37°C. 56 nM DNA containing both the toe-hold and a target sequence and 5.6 nM probe were incubated at 37°C, and samples were taken at indicated times and separated in a 3% agarose gel. Left: the Cy5 label (red) and Ethidium bromide staining (green) (panel a, composite), or via the Et.Br./Cy5 fluorescence separately (panel b and panel c). The part of the Cy5 channel that was used for the quantification in panel d) is marked with a colored box in panel c, where the color of the box corresponds with the color of the data in panel d). Next to the main target hybrid, several side bands form, indication multiple long-lived hybrids. The hybrids migrate slower through the gel as compared to the unbound target. This retardation increases when a higher percentage of agarose is used for the gel. Only the background corrected signal corresponding to the most prominent hybrid (blue squares) and to the free probe were used for the calculation of the K_D reported in the results.

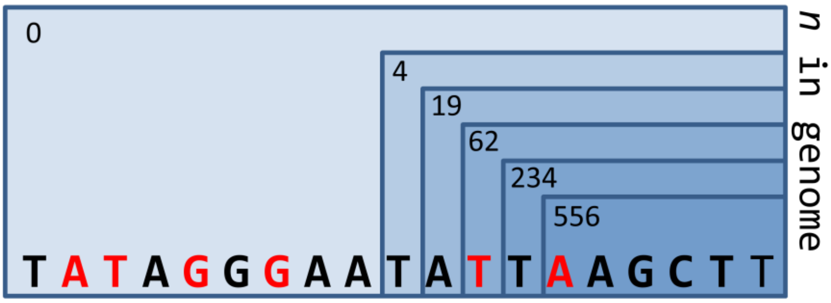

Figure S2: The number of sequences of complementary to the pLNA1 sequence in the *E. coli* genome quickly drops to 0 over the length of the recognition sequence. There are a total of 556 *Hind*III sites in the genome. The number of sequences compatible with a part of the probe is shown in each box. There is no target sequence with more than 10 complementary nucleotides in the *E. coli* genome.

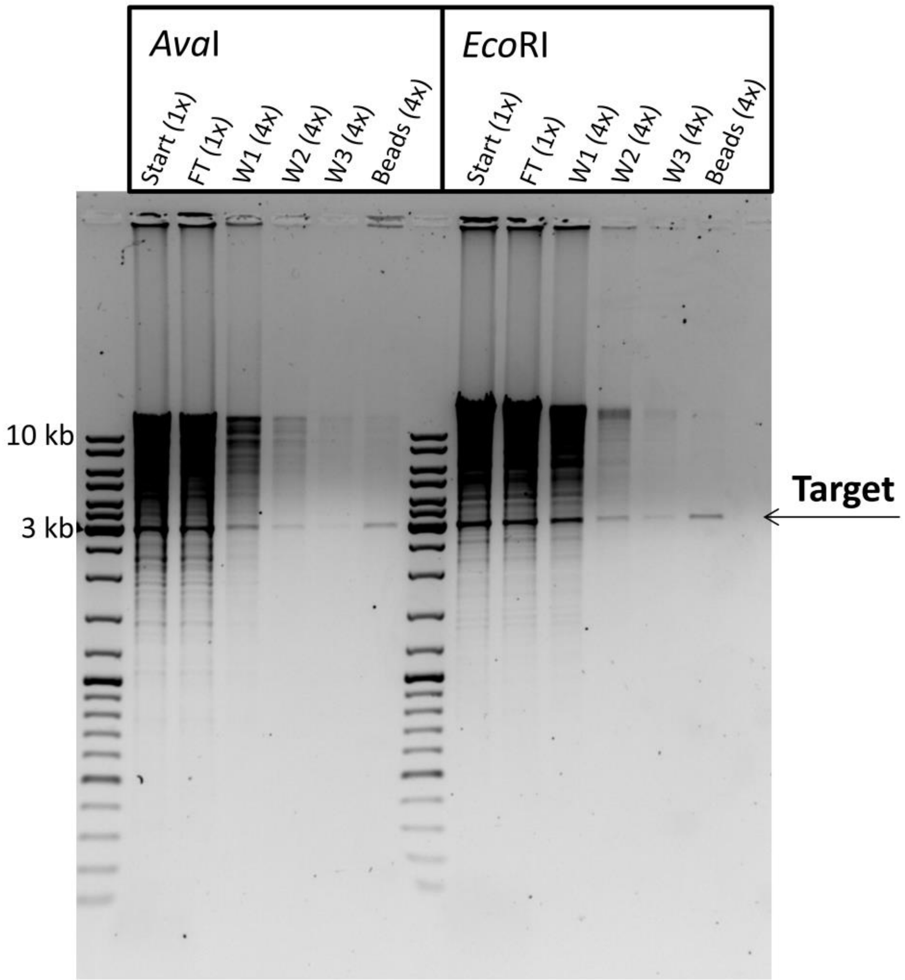

Figure S3: Purification of target DNA from a mixture of 100 ng target DNA mixed with 10 µg of *E.coli* chromosomal DNA using pLNA3. Genomic DNA (10 µg starting concentration) was digested with the restriction endonuclease *Ava*I, to create compatible toeholds, or with *Eco*RI to create incompatible toeholds. The genomic DNA was mixed with the target DNA and subjected to purification via the toehold LNA as described in Methods. DNA contained in samples from the starting material (Start), flow-through (FT), wash-step 1-3 (w1-3) and recovered from beads (beads) were separated in an agarose gel and visualized with EtBr. The relative fraction of the samples analyzed in the gel is indicated. Positions of selected marker bands in the first lane and the target are shown on the left and right, respectively. In both cases, the target was efficiently isolated.

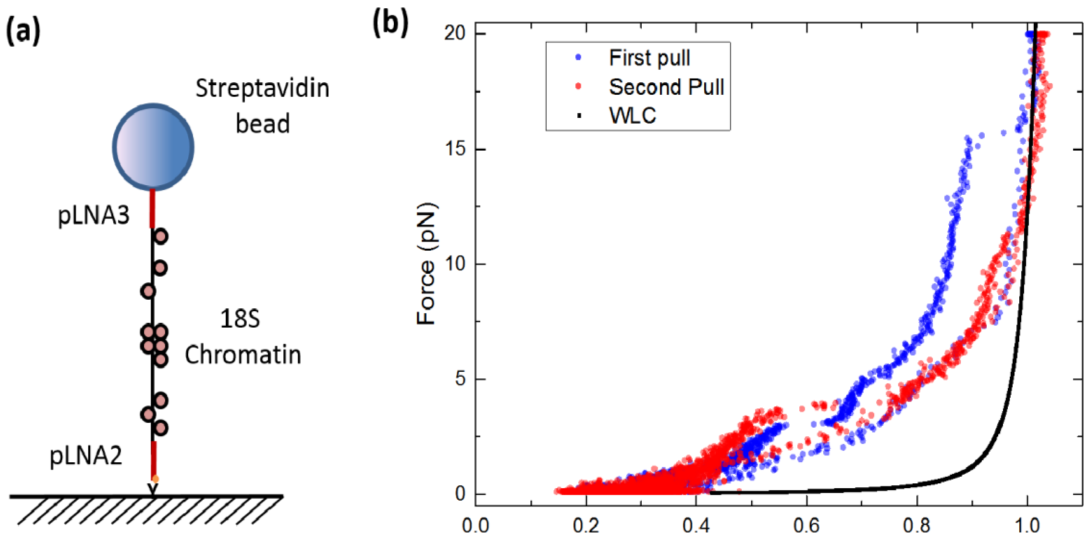

Figure S4: Partially purified chromatin fragments can be tethered between the surface and the paramagnetic beads using two LNA-toehold probes. (a) Two LNA probes were hybridized to the 18S chromatin fiber. pLNA2, tagged with digoxigenin, was used to immobilize the fragments to the surface of the coverslip and pLNA3, containing a biotin, was used to attach the fragment to the beads. (b) Since the LNA toehold probes can withstand forces up to 65 pN, multiple force extension experiments can be done on a single chromatin fragment.

Table S1, Sequences of target DNA substrates. The target sequence is red, the HindIII recognition site is underlined. The blunt ended target contains the target sequence, but lacks the toehold generated by *Hind*III in the Toehold target. For the targets restricted with *Hind*III, the fragment without target sequence is light grey.

	Sequence
Toehold target	gcgaagcgatgatttttgatctattaacagatatataaatgcaaaaactgcataaccactttaactaatactttcaacat<br>tttcggtttgtattacttcttattcaaatgtaataaaagtatcaacaaaaaattgttaatatacctctatactttaacgtca<br>aggagaaaaaaccccggatcggactactagcagctgtaatacgactcactatagggaatatta**agct**tggtacaaaa<br>atttttaaaaatttttgaattcacgtgtttaaatttataaaaatttcgagctcggatccactagtaacggccgccagtgtg<br>ctggaattctgcaga
Blunt end	gcgaagcgatgatttttgatctattaacagatatataaatgcaaaaactgcataaccactttaactaatactttcaacat<br>tttcggtttgtattacttcttattcaaatgtaataaaagtatcaacaaaaaattgttaatatacctctatactttaacgtca<br>aggagaaaaaaccccggatcggactactagcagctgtaatacgactcactatagggaatatta**agct**
Linear Target	Gcgaagcgatgatttttgatctattaacagatatataaatgcaaaaactgcataaccactttaactaatactttcaaca<br>ttttcggtttgtattacttcttattcaaatgtaataaaagtatcaacaaaaaattgttaatatacctctatactttaacgtc<br>aaggagaaaaaaccccggatcggactactagcagctgtaatacgactcactatagggaatatta**agctt**ggtacaaa<br>aatttttaaaaatttttgaattcacgtgtttaaatttataaaaatttcgagctcggatccactagtaacggccgccagtgt<br>gctggaattctgcaga
Negative Control	gctccaagctgggctgtgtgcacgaaccccccgttcagcccgaccgctgcgccttatccggtaactatcgtcttgagtc<br>caacccggtaagacacgacttatcgccactggcagcagccactggtaacaggattagcagagcgaggtatgtaggcg<br>gtgctacagagttcttgaagtggtggcctaactacggctacactagaaggacagtatttggtatctgcgctctgctgaa<br>gccagttaccttcggaaaaagagttggtagctcttgatccggcaaacaaaccaccgctggtagcggtggtttttttgttt<br>gcaagcagcagattacgcgcagaaaaaaaggatctcaagaagatcctttgatcttttctacggggtctgacgctcagt<br>ggaacgaaaactcacgttaagggattttggtcatgagattatcaaaaaggatcttcacctagatccttttaaattaaaa<br>atgaagttttaaatcaatctaaagtatatatgagtaaacttggtctgacagttaccaatgcttaatcagtgaggcacct<br>atctcagcgatctgtctatttcgttcatccatagttgcctgactccccgtcgtgtagataactacgatacgggagggctta<br>ccatctggccccagtgctgcaatgataccgcgagacccacgc
Figure S1 Target	cggtttgtattacttcttattcaaatgtaataaaagtatcaacaaaaaattgttaatatacctctatactttaacgtcaag<br>gagaaaaaaccccggatcggactactagcagctgtaatacgactcactatagggaatatta**agct**tggtacaaaaat<br>ttttaaaaatttttgaattcacgtgtttaaatttataaaaatttcgagctcggatccactagtaacggccgccagtgtgct<br>ggaattctgcaga
